# Effects of personality and rearing-history on the welfare of captive Asiatic lions (*Panthera leo persica*)

**DOI:** 10.7717/peerj.8425

**Published:** 2020-02-06

**Authors:** Sitendu Goswami, Praveen C. Tyagi, Pradeep K. Malik, Shwetank J. Pandit, Riyazahmed F. Kadivar, Malcolm Fitzpatrick, Samrat Mondol

**Affiliations:** 1 Wildlife Institute of India, Chandrabani, Uttarakhand, India; 2 Sakkarbaug Zoological Garden, Junagadh, Gujarat, India; 3 London Zoo, Zoological Society of London, London, United Kingdom

**Keywords:** Animal personality, Cognition, Ex-situ conservation, Behaviour diversity, Latency, Stereotypy, Captive animal welfare

## Abstract

**Background:**

The long-term success of ex-situ conservation programmes depends on species-appropriate husbandry and enrichment practices complemented by an accurate welfare assessment protocol. Zoos and conservation breeding programmes should employ a bottom-up approach to account for intraspecific variations in measures of animal welfare. We studied 35 (14:21) captive Asiatic lions in Sakkarbaug Zoological Garden, Junagadh, India to understand the implications of individual variations on welfare measures. We categorized the subjects based on personality traits (bold or shy), rearing history (wild-rescued or captive-raised), sex, and social-grouping. We explored the association of these categorical variables on welfare indices such as behavioural diversity, latency to approach novel objects, enclosure usage and aberrant repetitive behaviours. Further, we assessed the inter-relationships between different behavioural measures of welfare.

**Results:**

Our results show that intraspecific variations based on rearing-history and personality traits are significantly associated with the welfare states of captive Asiatic lions. Asiatic lions with bold personality traits (*M* = 0.50, *SD* = 0.12, *N* = 21) and those raised in captivity (*M* = 0.47, *SD* = 0.12, *N* = 16) used enclosure space more homogenously compared to shy (*M* = 0.71, *SD* = 0.15, *N* = 14) and wild-rescued (*M* = 0.67, *SD* = 0.15, *N* = 19) animals. Behaviour diversity was significantly higher in captive-raised (*M* = 1.26, *SD* = 0.3, *N* = 16) and bold (*M* = 1.23, *SD* = 0.26, *N* = 21) subjects compared to wild-rescued (*M* = 0.83, *SD* = 0.35, *N* = 19) and shy (*M* = 0.73, *SD* = 0.34, *N* = 14) individuals. Aberrant repetitive behaviours (stereotypy) were significantly lower in bold (*M* = 7.01, *SD* = 4, *N* = 21) and captive-raised (*M* = 7.74, *SD* = 5.3) individuals compared to wild-rescued (*M* = 13.12, *SD* = 6.25, *N* = 19) and shy (*M* = 16.13, *SD* = 5.4, *N* = 16) lions. Sex and social-grouping of subjects did not show significant associations with behavioural welfare indices. Interestingly, behaviour diversity was reliably predicted by the enclosure usage patterns and aberrant repetitive behaviours displayed by subjects.

**Discussion:**

Our findings underline the importance of individual-centric, behaviour-based, and multi-dimensional welfare assessment approaches in ex-situ conservation programmes. The results suggest that behavioural welfare indices complemented with individual variations can explain inter-individual differences in behavioural welfare measure outcomes of Asiatic lions. These findings also provide zoo managers with a non-invasive tool to reliably assess and improve husbandry practices for Asiatic lions. Understanding the unique welfare requirement of individuals in captivity will be crucial for the survival of the species.

## Introduction

Welfare defines a fine balance between pathophysiology and affective states, or the state of the animal as it copes with its environment (Broom, 1991; Spruijt, Bos & Pijlman, 2001; Meehan & Mench, 2007; Boissy et al., 2007; Butterworth, Mench & Wielebnowski, 2011; Panksepp, 2011). Modern welfare science advocates the creation of opportunities for animals to experience positive emotions (Fraser & Duncan, 1998; Fraser, 1999; Fraser, 2009; Dawkins, 2004; Whitham & Miller, 2016). Pro-welfare husbandry practices are vital for the biopsychosocial health of captive animals and long-term success of conservation breeding programmes (Hediger, 1968; Rabin, 2003; Broom, 2011). Studies show that animals housed under poor welfare conditions experience allostatic overload and chronic stress, that manifest as loss of behaviour diversity and cognitive abilities (Sheperdson, Carlstead & Wielebnowski, 2004; Mendl et al., 2009; Kroshko et al., 2016; Razal, Pisacane & Miller, 2016), ultimately reducing their survival and reproductive potential (Broom, 1991; Schreck, 2010). Ideally conservation breeding programs should conduct periodic welfare evaluations for the improvement of incumbent housing and husbandry practices, and realign with conservation goals (Engel, 1980; Korte, Olivier & Koolhaas, 2007; Broom, 2011; Moorhouse et al., 2015). In practice, ex-situ institutions continue to rely on unidimensional measures such as keeper ratings, physiological, and behavioural measures without accounting for individuality (Mason & Mendl, 2007; Boissy & Erhard, 2014; Chadwick, 2014). Intraspecific variations originating from personality (Locurto, 2006) and early-life experiences (Watters & Powell, 2012; Gartner, Powell & Weiss, 2016) determine the umwelt of individuals (Loehlin, 1992; Stamps & Groothuis, 2010), affective states (Harding, Paul & Mendl, 2004; Boissy & Erhard, 2014), and ultimately welfare (Carere & Locurto, 2011; Izzo, Bashaw & Campbell, 2011). Inter-individual differences in bold/shy personality traits (Mills, 1998; Gosling & John, 1999; Gartner, Powell & Weiss, 2016; Gartner & Powell, 2011; Gartner, Powell & Weiss, 2014) are associated with differential decision-making abilities (Carter et al., 2013), cognition (Morton, Lee & Buchanan-Smith, 2013; Griffin, Guillette & Healy, 2015) and coping responses (Wilson et al., 1993) to welfare deprivation (Koolhaas et al., 1999; Moneta & Spada, 2009; Goold & Newberry, 2017; Franks, Higgins & Champagne, 2014), and ultimately have a bearing on post-release fitness (Bremner-Harrison, Prodohl & Elwood, 2004). Early-life experiences can also have a bearing on the personality development of animals (Ainsworth & Bowlby, 1991; Higley et al., 1991; Loehlin, 1992; Frost et al., 2007; Stamps & Groothuis, 2010; Watters & Powell, 2012). Therefore, addressing early-life experiences and personality traits in welfare evaluation protocols can be vital to the success of ex-situ conservation breeding programmes (Wemelsfelder, 1997; Rabin, 2003; Reading, Miller & Shepherdson, 2013). Unfortunately, parameters of individuality are seldom addressed while designing housing and husbandry protocols for wild animals at conservation breeding programmes. Focused multi-species studies are required to understand how personality and early life-experiences (rearing-history) may be associated with behavioural welfare measures. Using captive Asiatic lions as a study system, we tried to understand the relationship between individual variations (viz., bold-shy traits, rearing history, sex, social grouping) with behavioural welfare measures.

The endangered Asiatic lion (*Panthera leo persica*) is now relegated to a fraction of its historic range, across scattered patches of the Greater Gir landscape of Gujarat, India (Banerjee et al., 2013). With a global wild and captive population of about 523 and 359 individuals (Srivastav, 2014; Pant, 2015), the future survival of Asiatic lions can be secured through a successful conservation-breeding program complemented by repatriation across historic ranges (Jhala et al., 2006; Meena, 2009). While extensive research on population ecology (Joslin, 1973; Jhala et al., 2009), behaviour (Meena, 2008), social dynamics (Meena, 2009; Chakrabarti & Jhala, 2017), and human-animal interaction (Joslin, 1973; Banerjee, Jhala & Pathak, 2010; Banerjee et al., 2013) of wild Asiatic lions has been conducted, the captive populations and their welfare needs have received relatively less attention. Pastorino et al. (2016) and Pastorino et al. (2017) studied feline-keeper interactions, personality variations, and behavioural aspects of welfare in captive Asiatic lions at London Zoo, but were limited by a small sample size (*N* = 4) and a short study period. There is a paucity of information on detailed welfare status of captive Asiatic lions despite a large ex-situ population spread among global zoological institutions. It is vital to standardize the welfare evaluation practices for this species to meet its long-term conservation goals. Since Indian ex-situ facilities account for more than 60 percent of the global captive Asiatic lion population (Srivastav, 2014), holistic welfare assessments at these sites can have a tangible impact on the conservation goals for the species.

We studied 35 Asiatic lions housed in the ex-situ conservation breeding centre of Sakkarbaug Zoological Garden (SZG), Gujarat, India to understand if rearing-history and personality (Allport & Allport, 1921) are important factors associated with intraspecific variations in behavioural welfare indices (Broom, 1986; Fraser, 2009). We categorized these subjects based on their rearing histories (wild-rescued and captive-raised), sex, social grouping (pair-housed and group-housed) and personality traits (bold and shy) (Wilson et al., 1994). We measured species-typical behaviour diversity (Powell, 1995; Wemelsfelder et al., 2000; Rabin, 2003; Clark & Melfi, 2012; Miller, Pisacane & Vicino, 2016; Watanabe, 2007; Skrzypczak, 2016; Owen, Swaisgood & Blumstein, 2017), space usage patterns (Kessel & Brent, 1996; Mallapur, Qureshi & Chellam, 2002; Ross & Shender, 2016), latency to novel objects (Murphy, 1977; Meehan & Mench, 2002; Sneddon, Braithwaite & Gentle, 2003), and proportion of aberrant behaviours (Mason & Rushen, 2006; Tan et al., 2013; Japyassú & Malange, 2014; Kroshko et al., 2016) understand the relationship between individual variations and welfare measures. We believe that this study will address knowledge gaps in animal welfare evaluation procedures, leading to the adoption of individual-focused husbandry and management practices at ex-situ endangered species conservation programmes.

## Materials & methods

### Research permit and ethical considerations

Research permit for this study was granted by the Gujarat Forest Department, India (Permit no: WLP/28/A/1316-21/2015-16). This study complies with the regulations of zoo animal welfare standards set by the Central Zoo Authority, Government of India.

### Study area

We conducted the study at Sakkarbaug Zoological Garden (SZG), which is situated within the natural range of Asiatic lions (*Panthera leo persica*). SZG is the coordinating zoo for the Asiatic lion conservation-breeding programme in India and hosts the largest captive population (*N* = 60) with the highest reported number of wild founders (Srivastav, 2014) The conservation breeding programme aims to stock a healthy population of captive Asiatic lions for possible repatriation to lost range habitats. The zoo has a separate off-display conservation breeding facility, which houses 47 Asiatic lions. A map (unscaled) of the off-display conservation breeding enclosures of SZG is provided in Fig. S1.

**Table 1:** Details of subjects included in the study, viz., house name, sex (M = Male, F = Female), origin (C = Captive, W = Wild), sex ratio for housing, Enclosure size, age in days, age class, and personality profiles.

Sl no	Subject name	Sex	Origin	Sex ration(M:F)	Enclosure size (m^2^)	Age in days	Space/animal	Age class	personality
1.	A1	M	W	1:1	1,300	3,414.00	650	old	shy
2.	Aftab	M	W	1:1	1,200	3,500.00	600	old	bold
3.	Amal	M	C	1:2	1,576	2,214.74	788	prime	bold
4.	Ambica	F	C	0:2	1,123	3,984.56	561.5	old	bold
5.	Amiya	F	C	0:2	1,123	2,037.56	561.5	prime	bold
6.	Ani	F	W	1:1	1,600	1,946.00	800	prime	shy
7.	Bahadur	M	C	1:2	1,600	653.33	533.33	sub	bold
8.	Bigtwin (Amrapur)	F	W	1:2	1,600	800.33	533.33	sub	shy
9.	Dharicub	M	W	1:2	1,100	619.50	366.6	sub	bold
10.	Dheer	M	W	2:0	1,600	5,389.08	800	old	bold
11.	Gina	F	C	1:1	1,100	1,316.90	550	prime	shy
12.	Girm	M	W	1:1	1,123	3,337.92	562.5	old	bold
13.	Hemal	M	W	1:2	1,700	2,029.00	566.6	prime	shy
14.	Hemali	F	W	1:2	1,271	2,149.00	635.5	prime	shy
15.	Jenifer	F	C	1:1	1,700	1,491.33	850	prime	bold
16.	Jesal	M	W	1:2	1,300	5,722.18	433.3	old	bold
17.	Maheswari	F	C	1:2	1,300	3,411.26	433.3	old	bold
18.	Mariyam	F	C	1:2	1,300	3,423.35	433.3	old	bold
19.	Maytri	F	C	1:1	1,123	3,072.90	562.5	old	bold
20.	Nagraj	M	W	1:2	6,542	3,227.00	2,180	old	shy
21.	Patvad	F	W	1:1	1,200	3,729.00	600	old	shy
22.	Patvadm	M	W	1:1	1,271	1,537.00	635.5	prime	shy
23.	Radha	F	C	1:2	1,576	1,291.30	525.3	prime	bold
24.	Rani	F	C	1:2	1,576	1,291.30	525.3	prime	bold
25.	Ranita	F	C	1:2	6,542	2,119.00	2,180	prime	bold
26.	Ranshi	F	W	1:2	6,542	5,109.00	2,180	old	shy
27.	Rudi	F	W	1:1	1,600	4,700.00	800	old	shy
28.	Smt (Amrapur)	F	W	1:2	1,600	800.33	533.3	sub	shy
29.	Subhi	F	C	1:1	1,700	2,240.33	850	prime	shy
30.	Sujan	F	W	1:1	1,300	2,790.80	650	prime	shy
31.	Taukir	M	C	1:1	1,303	2,448.00	651.5	prime	bold
32.	Teeta	F	C	1:2	1,700	2,166.93	566.6	prime	bold
33.	Tejaswini	F	C	1:2	1,700	4,320.93	566.6	old	bold
34.	Trakuda	M	W	1:2	1,700	3,956.93	566.6	old	bold
35.	Veer	M	W	2:0	1,600	5,387.30	800	old	bold

### Subjects and housing

We collected data from 38 (Male = 15, Female = 23) healthy Asiatic lions housed in the conservation breeding facility of SZG. During the study we removed three individuals (Male = 1, Female = 2) due to ongoing veterinary treatments, reducing our sample size to 35 individuals (Male = 14, Female = 21) (Table 1). The study subjects were either born in captivity (*N* = 16; Male = 3, Female = 13) or rescued from wild (*N* = 19; Male = 11, Female = 8). Individuals born in the zoo (*N* = 14) and rescued as cubs (*N* = 2) were categorized as captive-raised since they have similar life experiences. Lions that were rescued at a young age and spent most of their lives in captivity cannot have similar life-experiences as adult wild-rescued lions and hence were grouped in the captive-raised category. Most wild-rescued lions were rehabilitated as adults for treatment of injuries incurred due to infighting, and after making full recovery were assimilated in the conservation breeding programme. Some wild-rescued animals (*N* = 3) were rescued to ameliorate conflict caused by livestock depredation. All subjects were either housed in pairs (*N* = 17) or housed in a sex ratio of 1:2 (*N* = 18). All subjects (including the wild-rescued lions) were in socially cohesive groups and were housed in the same enclosure (with the same enclosure mates) for at least a year prior to the commencement of the study. This facility provided us with a unique opportunity to study the behaviour of wild-rescued and captive-raised lions under similar housing conditions.

Subjects were housed in 15 naturalistic enclosures spread across 8-ha area resembling the habitat of Asiatic lions. All enclosures were similar in design, devoid of enrichment devices, evenly populated with leafy trees (for shade and cover) and provided similar enclosure space per animal (400 m^2^), ensuring uniformity of housing conditions for all subjects. Due to the absence of complexity and an active enrichment intervention programme, all enclosures were deemed functionally barren to the subjects. The enclosure sizes ranged from 1,100–6,542 m^2^, with an average size of (M = 1,970 m^2^, SD = 1,685.24m^2^). Only one enclosure was 6,542 m^2^ in size, and most other enclosures were similar in sizes (M = 1,424 m^2^, SD = 224 m^2^). All enclosures included outdoor (paddocks) and indoor (retiring/feeding cells) areas (3 m × 3 m × 2 m dimensions) with continuous access to drinking water. Enclosure barriers consisted of v-shaped dry moats with walls at the proximal side and chain-linked fences with dual overhangs (4 m high) on the other three sides. Adjacent enclosures were separated by visual barriers in the form of dense bamboo thickets. Subjects were confined to feeding cubicles only during feeding time and had free access to all enclosure areas (including feeding cubicles) for the rest of the day. Subjects were fed separately at the indoor cubicles between 1,700–1,900 h six days a week, with a fast on Sundays. Subjects were fed in-house slaughtered and quality-inspected buffalo meat. The average meat consumption was 3.5 kg (SD = 0.5 kg) for females (*N* = 21) and 4.9 kg (SD = 1.4 kg) for males (*N* = 14). Most subjects were group-housed (1:2) (*N* = 18) or pair-housed (1:1) (*N* = 20). Four subjects were iso-sexually paired which included two male lions (2:0) and a mother-daughter dyad (0:2).

A group of animal keepers carried out all husbandry work for the subjects on a rotational basis, which meant that all subjects were accustomed to the same group of keepers. Since the conservation breeding area is off-display and restricts access to unauthorized personnel, subjects’ interactions with humans were limited to keeper interactions. The animal-keepers had trained the subjects to respond to their house names and vocal instructions for moving in and out of the feeding cubicles.

### Study design

We aimed to answer two broad research questions in this study; (a) how differences in bold/shy personality traits, rearing history, sex, and social grouping are associated with variations in behavioural welfare outcomes in a group of captive Asiatic lions? (b) How are the behavioural welfare indices (viz., enclosure usage, behaviour diversity, and aberrant repetitive behaviours) interlinked?

To answer the above questions, we categorized subjects and recorded outcomes of welfare measures. The detailed design for the study is given below.

### Personality assessment

We adopted a combination of keeper-rating and behaviour-coding techniques to reliably assess personality traits (Funder & Colvin, 1988; Funder, 1995; Gosling & Vazire, 2002; Highfill et al., 2010; Gartner & Powell, 2011). We separately interviewed three animal keepers with at least ten years of work experience to rate 38 subjects (15:23) on a scale of 1–9 (1-very low and 9- very high) for pre-selected bold (*N* = 10) and shy traits (*N* = 10) (Table S1). We found that all keepers agreed on their ratings for subjects (high inter-rater reliability, Cronbach’s alpha > 0.8). First, we averaged all keeper ratings (*N* = 3) subject-wise for all personality traits. Next, we calculated the average rating on bold (*N* = 10) and shy (*N* = 10) traits for each subject. Subjects that received an average score above seven on bold traits were categorized as bold, whereas an average rating above seven on shy traits were categorized as shy.

To validate keeper ratings for personality traits of subjects (bold/shy), we conducted novel-object tests in day kraals for ten minutes using video recorders in the absence of keepers and observers (Highfill et al., 2010). Naive observers (*N* = 3) with no prior exposure to study subjects, recorded the latency of subjects to interact with novel objects (Sih, Bell & Johnson, 2004; Frost et al., 2007) and calculated the percentage of bold vs shy behaviours (Powell & Svoke, 2008; Gartner & Powell, 2011; Corsetti et al., 2018) performed by the subjects during these tests (Tables S2 & S3). During these tests, the subjects were exposed to (a) unknown conspecifics, (b) unknown person and (c) non-food novel objects (lion-sam ball and bungee cord). All novel-object tests were conducted in an open-air day kraal adjacent to the paddock area of the enclosures. For the first test, we simultaneously released two subjects (same sex but unknown to one another) at adjacent day kraals and recorded their reactions to encountering a same-sex unknown conspecific. The latency counter was started as soon as both lions were released inside their respective day kraals. For the second test, we released the subject inside the day kraal and had a volunteer (unknown to the lion and not wearing a keeper’s uniform) approach the kraal, and stop at the median section facing the day kraal for ten minutes. The volunteer did not make any eye contact or vocal communication with the animal. The latency counter was started as soon as the volunteer reached the day kraal. For the final test, we placed a novel object (lion-sam ball or bungee cord) at the centre of the enclosure, and then released the subject inside the day kraal. The latency counter was started when the subject was released inside the day kraal. Observers used focal animal sampling (Altmann, 1974; Martin & Bateson, 1993) to calculate duration of all behavioural states and events performed by subjects during each of these tests. These focal observations were used to calculate the percentage of bold and shy behaviour performed by subjects (Table S3). We tested each subject separately to avoid confounding personality with dominance. We conducted the latency tests simultaneously for 12 individuals daily between 0900-1100 h. Since we did not want to overwhelm the animals with multiple novel stimuli on a single day, three sessions of novel-object tests were conducted for each subject on consecutive days. The novel object tests were conducted for ten minutes, and if subjects failed to approach the novel object after five minutes, they were categorized as shy. We repeated the novel object tests with unknown human and novel object after a month to check for trait consistency and calculated the average latency values for each subject. In the first session, lion-sam ball was used as the novel object which was replaced in the second session with a hanging bungee cord. The order of the latency tests was kept the same for all subjects. Three Asiatic lions undergoing veterinary treatments for physical injuries (Male = 1, Female = 2) showed inconsistencies in trait measures across different sessions. We excluded these animals from the study, thus reducing the number of subjects to 35 individuals (Male = 14, Female = 21). We found that keepers (*N* = 3) and observers (*N* = 3) reliably agreed on the personality type (Cronbach’s alpha > 0.9) of these 35 subjects. These subjects were further categorized based on rearing history (wild-caught = 19, captive-raised = 16), sex (Male = 14, Female = 21), and social grouping (pair-housed = 17, group-housed = 18).

### Behaviour data collection

For behaviour data collection, we used pre-existing ethograms for felids (Powell, 1995; Stanton, Sullivan & Fazio, 2015) and modified them to include unique behaviours displayed by subjects (Table 2). Two independent observers collected all behaviour data. To minimize inter-observer bias, behaviour recording was commenced after inter-observer reliability reached satisfactory levels from the same group of animals (Cronbach’s *α* > 0.9) (Caro et al., 1979; Gliem & Gliem, 2003). We recorded ten hour-long behaviour observation sessions (for four subjects) and found one-minute instantaneous scans (Altmann, 1974; Martin & Bateson, 1993; Amato, Van Belle & Wilkinson, 2013) were comparable to focal animal behaviour observation data (Altmann, 1974; Gilby, Pokempner & Wrangham, 2010; Amato, Van Belle & Wilkinson, 2013) in recording behavioural states and events for multiple subjects. We chose instantaneous scans as it provided a good balance between data-accuracy and observer fatigue. We recorded behaviour at three different time periods: 0500-1100 h, 1300–1800 h and 2200-0500 h in six-hour blocks. During each six-hour block, we conducted four one-hour sessions of instantaneous scans at one-minute intervals for one-hour duration followed by a 15-minute rest. During each scan, we recorded the behavioural state of the subject and its location in the enclosure. All occurrences of behaviour events were recorded separately. We used the frequencies of behavioural states and events to measure behaviour diversity of each subject during an observation session (one hour). We measured the directionality of all social interactions between subjects to gain a better understanding of the social cohesiveness of each enclosure group. We also video recorded behaviour observation sessions, which were used to fill any potential gaps in observer recording of instantaneous scans. We gathered a total of 2,009 h of behaviour observation data (average data of 57 h/subject) across 486 observation days. We collected information on the following behavioural welfare indices:

**Table 2:** Ethogram showing Asiatic lion behaviour (states and events) used in this study.

Behaviour class	Behavior	Description
Behavioural states	Locomotion	Walking or moving inside the enclosure.
	Rest	In a reclined position, head up or down.
	Sit	Haunches on the ground.
	Sleep	Reclined position, eyes closed.
	Climb	Climbing trees.
Discrete Behaviours	Defecate	Passing urine or fecal matter.
	Drink	Drinking water with distinct lapping sound.
	Groom	Lick or bite or scratch with paw self or conspecific.
	Lick	Running tongue over lips and nose multiple times in quick succession.
	Mark	Spraying object via perianal secretions or rubbing paws on the ground.
	Grab	Cautiously reaching or touching an object or conspecific with the forepaw in jabbing fashion.
	Roll	Body in the prostate position and rolling from side to side usually with the belly up.
	Rub	Pushing head or body against an object with head or part of the body.
	Scratch	Rubbing claws on an object (e.g. tree).
	Sniff	Inhaling scent from the air or an object.
	Vocalize	growling, roaring, grunting, humming, chuffing.
	Yawn	Opening mouth wide while showing canines and inhaling deeply.
	Stalk	Silently shadowing an object, conspecific, birds or keepers.
	Other	Any other behaviour observed.
Stereotypy	Pacing	Walking up and down on a fixed path occasionally raising its head to look up.
	Swaying	Subject moves head and body from side to side while standing next to a wall and shifting bodyweight from left to right foot.
	Head bobbing	Nodding head up and down while the animal is stationery.
	Nose rubbing	Subject rubs nose on enclosure wall or enclosure barrier continuously without any aim or purpose.

**Notes.**

* Ethogram showing Asiatic lion behaviour (states and events) used in this study.

#### Enclosure usage

**Figure 1:**
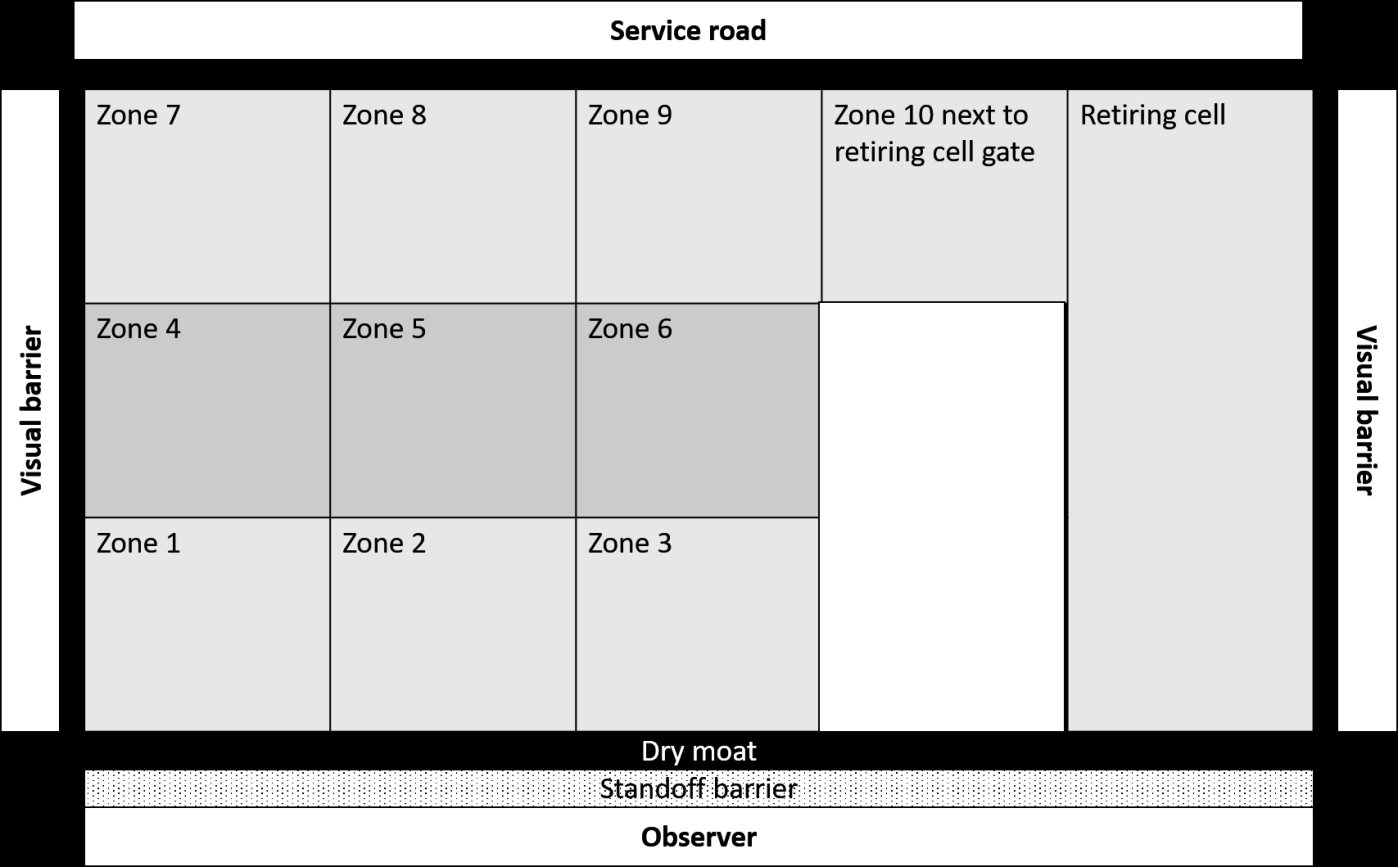
Schematic representation of an enclosure in Sakkarbaug zoological garden with the layout of zones for behavioural observations of enclosure use by study subjects.

Enclosure use is a critical behavioural parameter that is influenced by the biological relevance of different zones of the captive environment (Traylor-Holzer & Fritz, 1985; Plowman, 2003; Rose & Robert, 2013; Ross & Shender, 2016). Homogenous usage is indicative of a complex and novel enclosure design (Ross & Shender, 2016; Mallapur, Qureshi & Chellam, 2002; Rose & Robert, 2013) which are considered more important drivers of welfare than enclosure area (Traylor-Holzer & Fritz, 1985). We divided each enclosure into ten equal zones, which included three broad zones viz. (a) proximal, (b) medial, and (c) distal zones. Each of these broad zones was further subdivided into three smaller zones as (i) left, (ii) middle, and (iii) right. The tenth zone was the paddock area next to the retiring cell (Fig. 1). We recorded the enclosure zone location of subjects during each scan. We calculated the spread of participation index (SPI) (Plowman, 2003) of enclosure usage for all 35 subjects across 486 observation days using instantaneous scan data. We calculated the SPI values using the following formula:

SPI= $ \frac{\sum {|}fo-fe{|}}{2 \left( N-femin \right) } $

where f_o_ stands for the observed frequency of usage of enclosure zones, f_e_ stands for the expected frequency of enclosure usage. N stands for gross observations in all zones of the enclosure and f_emin_ stands for the expected frequency of observation for the smallest zone (Plowman, 2003). SPI measures indicate the homogeneity of space usage. A high SPI value (close to 1) indicates that subjects are biased towards certain areas of the enclosure, while a lower SPI value (close to 0.5 or lower) indicates that lions use most areas of the enclosure equitably.

It is noteworthy to point out that social animals like lions are likely to have hierarchies and dominant animals are likely to monopolize preferred areas, but an ideal enclosure should provide equal opportunities for exploration and free movement to all individuals. In this study, we aimed to measure the enclosure zone usage pattern of each subject in a social configuration to ascertain how it met individual welfare requirements and related to other welfare indices.

#### Species-typical behaviour diversity

Behaviour diversity is indicative of the scope of novelty, and complexity afforded to animals in captivity (Wemelsfelder et al., 2000; Haskell et al., 2018). Maintaining behaviour diversity of captive animals housed at breeding programmes is essential for the preservation of essential learned behaviours required for post-release survival (Rabin, 2003). Complex and cognitively enriching enclosures have been shown to stimulate captive animals to display a diverse behaviour repertoire (Spiezio et al., 2018). We used Shannon-Weiner diversity index (SWI) to measure species-typical behaviour diversity as this approach considers both richness and evenness of species-typical behaviours in the data set (Clark & Melfi, 2012; Miller, Pisacane & Vicino, 2016; Spiezio et al., 2018). We compiled an ethogram of all behaviour states and events observed from all subjects during the study period (Table 2). We pooled all behaviour observations of each subject to calculate behaviour diversity. We excluded aberrant repetitive behaviours from the calculations since they did not qualify as species-typical behaviours.

#### Aberrant repetitive behaviours (ARB)

Aberrant repetitive behaviours (ARB) are reliable measures of poor welfare conditions (Dawkins, 2004; Watters, 2009; Kroshko et al., 2016) as they are precursors of cognitive dysfunction and neurophysiological changes (Muehlmann & Lewis, 2012). For this study, we measured the proportion of scans spent by each subject performing ARBs as an indicator of poor welfare (Mason & Latham, 2004). ARBs mostly included, stereotypic swaying, pacing, and nose rubbing behaviours (Table 2). We did not record anticipatory displacement behaviours, for example, pacing before feeding (Watters, 2014) or during interaction with conspecifics as ARB. We considered behaviours persisting over five consecutive scans and without an observer-discernible cause as ARB. Therefore, displacement behaviours performed before feeding time, or in response to keeper activities were not considered as aberrant repetitive behaviours.

### Data analysis

We tested the following hypotheses in this study:

1. There would be no variations in behaviour indices between male and female subjects and the measures would perform uniformly for both sexes.
2. The wild-rescued lions would display higher behaviour diversity than captive-raised animals.
3. Bold individuals would differ in behavioural welfare indices compared to shy individuals.
4. There would be no difference in behavioural welfare parameters between pair-housed and group-housed subjects.
5. There will be no significant prediction of behavioural diversity by the proportion of aberrant repetitive behaviour and the enclosure usage patterns of subjects.

We used R statistical software version 3.4 and 3.5.2 through RStudio (RStudio, 2015) using packages, dplyr (Wickham, 2016), ggplot2 (Wickham, 2016), lubridate (Spinu, Grolemund & Wickham, 2018), tidyverse (Wickham, 2017), and funModeling (Casas, 2019). For exploratory data analyses, we used Shapiro–Wilk test and Levene’s test to ascertain the normal distribution and homogeneity of variance in SPI, SWI, latency and ARB values, respectively. We conducted bivariate Pearson’s correlation to ascertain the strength of association between four above-mentioned welfare indices. We also checked for correlation between enclosure area and zone usage bias.

For statistical analysis, we had four categorical predictor variables (personality trait, rearing history, sex, and social grouping), each with two levels (viz. bold & shy, wild-rescued & captive-raised, male & female and pair-housed & group-housed). We compared welfare indices across groups (categorical predictor variables) using independent samples *t*-tests for normally distributed dependent variables (enclosure usage, behaviour diversity, and ARBs). We used non-parametric Kolmogorov–Smirnov test for latency measures and the proportion of bold and shy behaviours performed by subjects during the novel object test. When comparing between means of two groups with different sample sizes, it is important to report effect sizes in addition to p-values to indicate the scale-independent degree of difference. We calculated effect sizes to quantify differences in welfare measures between groups (Cohen, 1992; Lakens, 2013).

Finally, we conducted multiple regression analysis to understand how the behaviour diversity of captive animals were predicted by their enclosure usage patterns and ARB levels. Before conducting a regression analysis, we checked for multicollinearity between independent variables using measures of VIF (variance inflation factor). Since enclosure usage and ARBs were not highly correlated, we used them as predictors for behaviour diversity in regression analysis.

## Results

### Validation of keeper ratings

**Figure 2:**
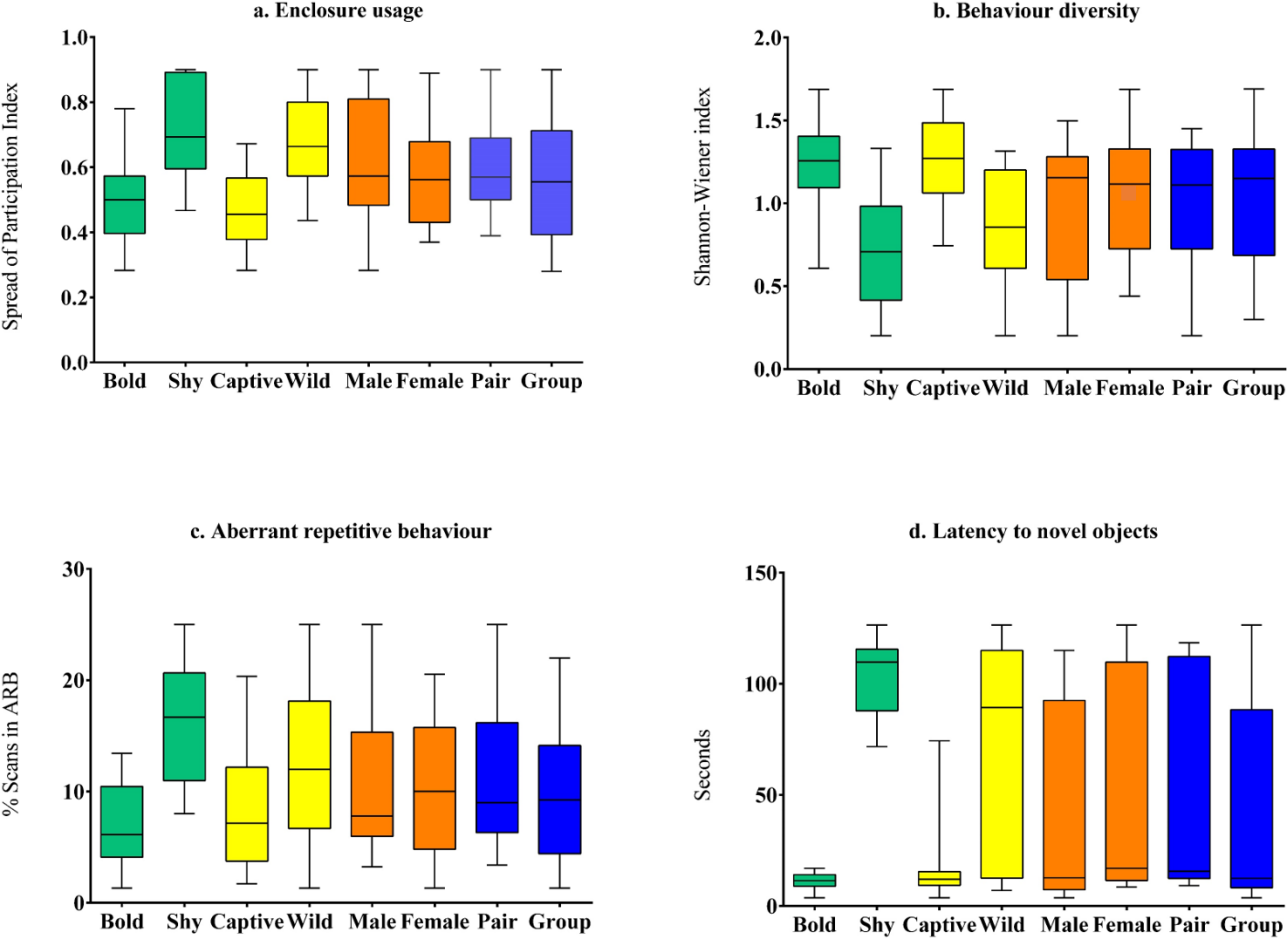
Comparison of behavioural welfare indices of Asiatic lions across personality (bold and shy), life-history (wild and captive), sex (male and female), and social grouping (pair-housed vs group-housed) categories. The behavioural welfare indices used here are (A) Enclosure usage; (B) Behaviour diversity; (C) Aberrant repetitive behaviour; and (D) Latency to novel objects.

The mean latency times for all subjects after averaging both trials was 47.76 s (SD = 46.85). Subjects categorized as bold by keepers (*M* = 11.13, SD = 3.65, *N* = 21) showed significantly lower latency values (*z* = 2.89, *p* < 0.01, Cohen’s *d* = 7.28) compared to subjects categorized as shy (*M* = 102.71, SD = 17.4, *N* = 14)(Table 3, Fig. 2). Bold subjects also showed significantly higher percentage of bold behaviours (*M* = 87.24, SD = 8.74) than shy individuals (*M* = 15.86, SD = 9.5) (*t*(33) =  − 10.57, *p* < 0.01) (Table S3). These results validate the keeper rating of subjects on the bold-shy scale.

**Table 3:** Comparison of welfare indices (viz., enclosure usage, behaviour diversity, aberrant behaviours, and latency to novel objects) between Asiatic lions of different categories (captive-raised vs wild-rescued, bold vs shy, male vs female, and pair-housed vs group- housed).

Life-histories	**Captive-raised**	**Wild-rescued**	**t(33)**	***p***-**value**	**Effect size (Cohen’s d)**
Enclosure usage	0.47 ± 0.12	0.67 ± 0.15	4.28	<0.000	1.47
Behaviour diversity	1.26 ± 0.3	0.83 ± 0.35	−3.94	<0.00	1.35
Aberrant behaviours	7.74 ± 5.3	13.12 ± 6.25	2.71	0.10	0.92
Latency to novel object	18.61 ± 21.55	72.30 ± 48.7	2.89^*^	0.000	1.42
**Personality**	***Bold***	***Shy***	**t(33)**	***p***-**value**	***Effect size (Cohen’s d)***
Enclosure usage	0.5 ± 0.12	0.71 ± 0.15	−4.572	<0.000	1.54
Behaviour diversity	1.23 ± 0.26	0.73 ± 0.34	4.897	<0.000	1.64
Aberrant behaviours	7.01 ± 3.9	16.13 ± 5.4	−5.825	<0.000	1.94
Latency to novel object	11.13 ± 3.65	102.71 ± 17.4	−4.95^*^	<0.000	7.28
**Sex**	***Male***	***Female***	**t(33)**	***p***-**value**	***Effect size (Cohen’s d)***
Enclosure usage	0.61 ± 0.2	0.57 ± 0.15	5.28	0.60	0.17
Behaviour diversity	0.96 ± 0.43	1.1 ± 3.5	−0.85	0.4	0.28
Aberrant behaviours	11.04 ± 7.05	10.41 ± 6.02	0.282	0.78	0.09
Latency to novel object	37.02 ± 45	54.91 ± 47.8	−1.11^*^	0.27	0.38
**Social grouping**	**Pair housed** (***n = 17***)	**Group housed** (***n = 18***)	**t(33)**	***p***-**value**	**Effect size (Cohen’s d)**
Enclosure usage	0.6 ± 0.13	0.56 ± 0.19	−0.69	0.49	0.22
Behaviour diversity	0.99 ± 0.33	1.06 ± 0.42	0.46	0.64	0.18
Aberrant behaviours	11.33 ± 6.12	10.02 ± 6.69	−0.6	0.55	0.2
Latency to novel object	55.14 ± 48.2	40.78 ± 45.81	0.95^*^	0.31	0.3

**Notes.**

* *Z*values from Kolmogorov Smirnov test for independent samples.

#### Comparison of welfare measures across categorical independent variables

#### Latency to novel objects

Captive-raised individuals (*M* = 18.61, SD = 21.55, *N* = 16) displayed significantly (*z* = 1.86, *p* = 0.02, Cohen’s *d* = 1.42) lower latency compared to wild-rescued individuals (*M* = 72.30, SD = 48.7, *N* = 19) (Table 3, Fig. 2). We found no difference in latency scores between male (*M* = 37.02, SD = 45, *N* = 14) and female (*M* = 54.9, SD = 47.8, *N* = 21) subjects (*z* = 0.89, *p* = 0.39, Cohen’s *d* = 0.38). Latency values did not vary significantly between pair-housed (*M* = 55.14, SD = 48.2, *N* = 17) and group-housed (*M* = 40.78, SD = 45.81, *N* = 18) lions (*z* = 0.9, *p* = 0.31, Cohen’s *d* = 0.3).

#### Enclosure usage

Enclosure space usage patterns varied significantly between subjects with different personality types and rearing histories. Wild-rescued individuals used enclosure space less homogeneously (*M* = 0.67, SD = 0.15, *N* = 19) than captive-raised individuals (*M* = 0.47, SD = 0.12, *N* = 16) (t (33) = 4.28, *p* < 0.01, Cohen’s *d* = 1.47) (Table 3, Fig. 2). Subjects with bold personality traits showed significantly less enclosure-zone bias (*M* = 0.5, SD = 0.12, *N* = 21) compared to individuals with shy traits (*M* = 0.71, SD = 0.15, *N* = 14) (*t*(33) =  − 4.572, *p* < 0.01, Cohen’s *d* = 1.54). Overall, the SPI value of males (*M* = 0.61, SD = 0.20, *N* = 14) was not significantly different from female (*M* = 0.57, SD = 0.15, *N* = 21) lions (t (33) = 5.28, *p* = 0.6, Cohen’s *d* = 0.17). The enclosure usage patterns of group-housed (*M* = 0.56, SD = 0.19, *N* = 18) and pair-housed subjects (*M* = 0.60, SD = 0.13, *N* = 17) (*t*(33) =  − 0.69, *p* = 0.49, Cohen’s *d* = 0.22) were similar.

#### Species-typical behaviour diversity

We found that species-typical behaviour diversity of captive-raised animals (*M* = 1.26, SD = 0.3, *N* = 16) was significantly higher than wild-rescued animals (*M* = 0.83, SD = 0.35, *N* = 19) (*t*(33) =  − 3.94, *p* < 0.01, Cohen’s *d* = 1.35) (Table 3, Fig. 2). Further, bold subjects displayed higher behaviour diversity (*M* = 1.23, SD = 0.26, *N* = 21) than shy individuals (*M* = 0.73, SD = 0.34, *N* = 14) (*t*(33) = 4.89, *p* < 0.01, Cohen’s *d* = 1.64) (Table 3, Fig. 2). Behaviour diversity levels were similar between male (*M* = 0.96, SD = 0.43, *N* = 14) and female (*M* = 1.1, SD = 0.35, *N* = 21) lions (*t*(33) =  − 0.85, *p* = 0.4). Group-housed (*M* = 1.06, SD = 0.42, *N* = 18) and pair housed subjects (*M* = 0.99, SD = 0.33, *N* = 17) showed similar levels of behaviour diversity (*t*(33) = 0.64, *p* = 0.64, Cohen’s *d* = 0.18).

#### Aberrant repetitive behaviours (ARB)

Wild-rescued individuals (*M* = 13.12, SD = 6.25, *N* = 19) expressed higher proportions of ARBs than captive-raised individuals (*M* = 7.74, SD = 5.3, *N* = 16) (*t*(33) = 2.71, *p* = 0.01, Cohen’s *d* = 0.92) (Table 3, Fig. 2). Bold individuals (*N* = 21) showed significantly lower levels of stereotypic behaviour such as pacing and swaying (*M* = 7.01, SD = 4, *N* = 21) compared to shy individuals (*M* = 16.13, SD = 5.4, *N* = 14) (*t*(33) =  − 5.82, *p* < 0.01, Cohen’s *d* = 1.94) (Table 3, Fig. 2). We found no difference in the expression of ARBs between male (*M* = 11.04, SD = 7.05, *N* = 14) and female (*M* = 10.41, SD = 6.02, *N* = 21) subjects (*t*(33) = 0.282, *p* = 0.78, Cohen’s *d* = 0.09), as well as between group-housed (*M* = 10.02, SD = 6.69, *N* = 17) and pair-housed subjects (*M* = 11.33, SD = 6.12, *N* = 17) (t(33)=-0.6, *p* = 0.55, Cohen’s *d* = 0.2).

### Inter-relationship between welfare indices

Latency was positively correlated (Fig. S2, Table 4) to enclosure zone bias (*r* = 0.67, *N* = 35, *p* < 0.01), and proportion of ARBs (*r* = 0.70, *N* = 35, *p* < 0.01). Latency was negatively correlated to behaviour diversity (r=−0.67, *N* = 35, *p* < 0.01). Enclosure zone bias was positively correlated with latency values (*r* = 0.66, *p* < 0.01), and proportion of ARBs (*r* = 0.66, *p* < 0.01) (Fig. S2, Table 4), but was negatively correlated with behaviour diversity (*r* =  − 0.71, *p* < 0.01). We found that enclosure zone bias was weakly positively correlated to enclosure size (*r* = 0.36, *p* = 0.05). Behaviour diversity was negatively correlated with latency to novel objects, (*r* =  − 0.67, *p* = 0.01), ARBs (*r* = 0.91, *p* = 0.01), and enclosure usage (*r* =  − 0.71, *p* = 0.01) (Supplementary Fig. 2, Table 4). ARB was positively correlated with latency to novel objects (*r* = 0.70, *p* = 0.01), and enclosure usage (*r* = 0.66, *p* = 0.01) but was negatively correlated with behaviour diversity (*r* =  − 0.91, *p* = 0.01) (Supplementary Fig. 2, Table 4). Multiple regression analysis (Table 5) indicated that ARBs and space usage homogeneity explained 85% of the variance in the behaviour diversity (*R*^2^ = 0.85, *F*(2, 32) = 90.92, *p* < 0.01) (Table 5). The predicted regression equation is Behaviour diversity =1.8 + (−0.046)*x*(*ARB*) + (−0.46)*x* (Enclosure usage). The results from the regression indicate that subjects that show less ARB and use enclosure space more homogenously are likely to have higher behaviour diversity.

**Table 4:** Pearsons bivariate correlations between behavioural welfare indices, age of subjects and enclosure size.

	Enclosure usage	Behaviour diversity	Aberrant behaviours	Latency to novel object	Age	Enclosure size
Enclosure usage						
Behaviour diversity	−0.71^***^					
Aberrant repetitive behaviours	0.66^***^	−0.91^***^				
Latency to novel object	0.66^***^	−0.67^***^	0.70^***^			
Age	0.26	0.04	−0.19	−0.09		
Enclosure size	0.36	−0.3	0.26	0.16	0.14	

**Notes.**

*** *p* < 0.001.

** *p* < 0.01.

* *p* < 0.05.

**Table 5:** Multiple regression results for inter-relationships between behavioural welfare measures.

**Independent variables**	**Estimates**	**Std error**	***t***	***p* values**	***R*-squared**	**F**	**Durbin-Watson**
Behaviour diversity							
(Intercept)	1.793	0.09	18.870	2e−	0.85	90.92	1.78
Enclosure space usage	−0.459	0.20	−2.258	0.030			
Aberrant repetitive behaviour	−0.046	0.005	−8.49	1.04e−09			

## Discussion

To the best of our knowledge, this is the first empirical study to showcase the association of personality traits in wild-rescued and captive-raised Asiatic lions across multiple behavioural welfare measures. Our sample size constitutes 10% of the global captive stock of Asiatic lions, making the results relevant for the global conservation initiatives for this species. Several studies have asserted the importance of multiple indices for welfare assessment at zoos and conservation breeding programmes viz., behaviour diversity (Powell, 1995; Clark & Melfi, 2012), enclosure usage (Ross & Shender, 2016; Kistler et al., 2010) and stereotypy in captive animals (Dawkins, 2004; Kroshko et al., 2016; Clegg, 2018). However, most ex-situ institutions continue to use uni-dimensional measures to assess welfare and seldom address individuality (Van der Harst & Spruijt, 2007; Volpato et al., 2009; Hill & Broom, 2009; McMahon et al., 2013) . We addressed this issue by showcasing the importance of an individual-focused multi-dimensional approach to welfare assessments. Overall, Asiatic lions with different personality traits (bold and shy) and rearing-history (captive-raised and wild-rescued) differed significantly on measures of welfare, which supports earlier studies linking animal welfare with individuality (Carere & Locurto, 2011; Gartner & Powell, 2011; Boissy & Erhard, 2014; Gartner, Powell & Weiss, 2016). We did not observe any sex-specific variations in behavioural welfare measures of the subjects, confirming our first hypothesis. Contrary to our second hypothesis, wild-rescued lions showed low behaviour diversity, high enclosure-use bias, increased stereotypy and higher latency to novel objects compared to captive-raised subjects. Our results challenge existing research findings that report wild-rescued animals to be less likely to develop stereotypies than captive-raised individuals, while not accounting for animal personality (Cooper & Nicol, 1996; Schoenecker, Heller & Freimanis, 2000). It is possible that such pattern in our study is driven by higher proportion of shy individuals (*N* = 12) in the wild-rescued category compared to the captive-raised subjects (*N* = 2). Nevertheless, these results clearly show that wild-rescued lions may not necessarily be at a better state of welfare by default when compared to captive-raised individuals under similar housing conditions. Further empirical studies with equal sampling across bold and shy continuum between captive-raised and wild-rescued individuals are required to confirm these patterns. Our results supported the third hypothesis that lions with bold personalities are more resilient to functionally barren housing conditions than shy subjects, which supports earlier studies (Cole & Quinn, 2014; Japyassú & Malange, 2014). Moreover, present welfare assessment protocols often do not consider individual requirements as modifiers for species-specific husbandry practices. The effects of animal personality on welfare (Izzo, Bashaw & Campbell, 2011; Coelho, De Azevedo & Young, 2012; Razal, Pisacane & Miller, 2016) and its implications for post-release survival (Bremner-Harrison, Prodohl & Elwood, 2004; Watters, 2009) are well documented. This study aligns with the conservation goals for Asiatic lions by addressing individuality in welfare assessment (Rabin, 2003; Izzo, Bashaw & Campbell, 2011; Coelho, De Azevedo & Young, 2012; Razal, Pisacane & Miller, 2016). Group-housed and pair-housed subjects were similar across all behavioural welfare indices, supporting our fourth hypothesis. Although the enclosures were aesthetically pleasing, appropriate in terms of size, naturalistic vegetation and social grouping of animals; the abject lack of multisensory stimulation in terms of enrichment and novel experiences rendered them functionally barren to the subjects (Reading, Miller & Shepherdson, 2013). In this study we measured the evenness of enclosure use, which represents the functional space of the enclosure rather than available space. We found that small variations in enclosure sizes do not associate with significant shifts in behavioural welfare of Asiatic lions, which is in line with previous studies that place more importance on enclosure design (Tan et al., 2013), complexity and species-appropriateness than enclosure area (Rose & Robert, 2013; Herrelko, Buchanan-Smith & Vick, 2015; Neal Webb, Hau & Schapiro, 2018). The correlation between enclosure size and space usage bias was weak but positive, which means that increasing enclosure sizes were associated with higher zone-usage bias. This underlines the urgent need to provide complex captive environments that promote homogenous space usage and stimulate expression of species-typical behaviours.

Bivariate correlations and regression model presented in this study underline strong inter-linkages between behaviour diversity, enclosure usage and ARBs (Rabin, 2003; Melotti et al., 2011; Rose & Robert, 2013; Kroshko et al., 2016). Our results provided evidence that behaviour diversity can be explained by level of ARB and space usage patterns, which is in line with findings from existing studies (Wemelsfelder et al., 2000; Watters, Margulis & Atsalis, 2009; Clark & Melfi, 2012). From our results it can be surmised that subjects that are under less stress (low ARB) are likely to show homogenous enclosure space usage and a diverse behaviour repertoire and vice versa. Zoo managers must pay close attention to the development of high enclosure-zone biases (Ross & Shender, 2016) conjugated with low behaviour diversity (Clark & Melfi, 2012; Rose & Robert, 2013) as that may develop into severe levels of ARBs (Konjević et al., 2015). Overall, these findings indicate that behavioural welfare measures (enclosure usage, behaviour diversity, and ARB) have strong interlinkages and vary across inter-individual differences (viz., personality, rearing-history, sex, and social grouping). Zoo managers must take a proactive approach to improve the welfare status (Mendl et al., 2009) of captive Asiatic lions. Since enrichment interventions are effective in bringing complexity to sterile enclosures (Swaisgood & Shepherdson, 2005; Reading, Miller & Shepherdson, 2013), tailored-enrichment interventions must be integrated with husbandry practices for Asiatic lions (Powell, 1995; Cannon et al., 2016). Studies also show that positive keeper-animal relationships can improve welfare (Whitham & Wielebnowski, 2013). Finally, regular behaviour monitoring of captive wild animals should be incorporated into the husbandry practices to improve welfare and prevent development of stereotypy (Watters, Margulis & Atsalis, 2009).

## Conclusion

Felids are among the most represented taxa across zoological institutions, which necessitates an effective and holistic welfare evaluation protocol (Szokalski, Litchfield & Foster, 2013). Our findings underline the importance of individual-tailored husbandry design (Boissy & Erhard, 2014) to promote animal welfare at conservation breeding centers (Dawkins, 1990; Fraser & Duncan, 1998; Bateson & Matheson, 2007; Fraser, 2009; McMahon et al., 2013). Our results highlight that personality traits, enclosure usage patterns, behavioural diversity and stereotypy measurement as cost-effective and non-invasive tools that can reliably diagnose welfare needs in captive wild animals (Broom, 1991; Mason & Mendl, 2007) and conduct post-occupancy evaluations of enclosures (Wilson et al., 2003). These assessments can also help in effective management of endangered species through personality-matched pairings for breeding success (Fox & Millam, 2014; Martin-Wintle et al., 2017), and profiling of individuals most suited for repatriation (Bremner-Harrison, Prodohl & Elwood, 2004; Watters, 2009).

More specifically for Asiatic lions, Indian and Southeast Asian zoos account for more than 60% of the global captive Asiatic lion population (Srivastav, 2014). Unlike many European or North American zoological institutions most Indian zoos are state-funded and follow husbandry guidelines delineated by governmental animal-welfare agencies. Current governmental policies and guidelines for managing captive wild animals in Indian zoos do not explicitly consider inter-individual variations in animal welfare practises. Even existing studies on captive African (Powell, 1995; Watters & Powell, 2012) and Asiatic lions (Pastorino et al., 2016; Pastorino et al., 2017) have not translated into tangible shift in ongoing husbandry practises. Our findings provide strong scientific evidence that can lead to a paradigm shift in Government policies towards animal management in the Indian zoos and the global conservation breeding programmes. These results will be crucial to the large-scale uptake of individual-focused welfare assessment practices at Indian zoos. Such policy-level changes to animal welfare guidelines will strengthen ex-situ conservation practices in this region. Future cross-institutional studies on how internal (physiology) or external factors (enrichment interventions) interact with personality to predict welfare outcomes can shed light on some of the trends highlighted in this study. We hope that this study encourages managers and biologists to revisit traditional husbandry protocols and change them to meet the cognitive needs of individual animals under their care.

##  Supplemental Information

10.7717/peerj.8425/supp-1 Figure S1 Schematic layout of enclosures at Asiatic lion conservation breeding center, Sakkarbaug Zoological Garden The study was conducted at open-air enclosures from A1-A15. Enclosures A13, and A12 were empty and enclosure marked Cheetah housed another species and were excluded from the study.

10.7717/peerj.8425/supp-2 Figure S2 Scatterplot matrix (lower diagonal) with bivariate correlational analysis (upper diagonal) of various welfare indices viz., Enclosure usage, Behaviour diversity, Aberrant repetitive behaviour (ARB), Latency, and age of subjects The ellipsoids inside each scatterplot represent 50% concentration of data points. The upper diagonal box represents correlation coefficients between welfare indices with significance values ^∗^0.05, ^∗∗^0.01, ^∗∗∗^0.001.

10.7717/peerj.8425/supp-3 Table S1 Personality traits, behaviour details and Novel object test results

10.7717/peerj.8425/supp-4 Supplemental Information 4 Raw data for all parameters from 35 Asiatic lions

## Additional Information and Declarations

### Data Availability

The following information was supplied regarding data availability: Details of the housing areas and conditions for the captive subjects are available in the Study Area section of the article. Related schematic diagrams are available in Fig. 1 and Fig. S1. All details related to the individual subjects, their personality and bold/shy behaviour details are available in Table 1. The ethogram used in this study is available in Table 2. The compiled behaviour data used for all analyses is available in Data S1. No additional data is required to replicate the results presented in this study.
