# Supplementary figures and images for: Effects of personality and rearing-history on the welfare of captive Asiatic lions (Panthera leo persica)
Source: PeerJ. 2020 Feb 6;8:e8425. doi: 10.7717/peerj.8425 (PMC7007979; doi:10.7717/peerj.8425)

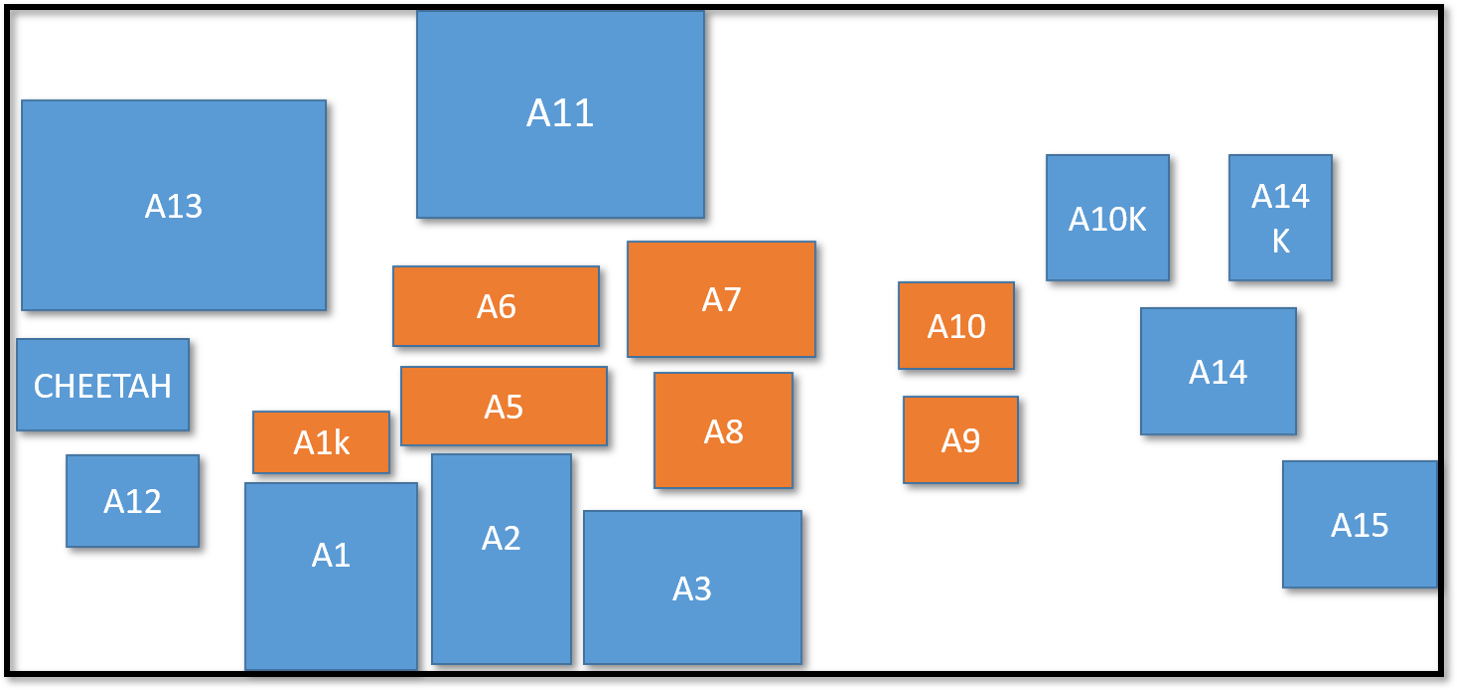

Supplement: Figure S1 — The study was conducted at open-air enclosures from A1-A15. Enclosures A13, and A12 were empty and enclosure marked Cheetah housed another species and were excluded from the study. [file peerj-08-8425-s001.png]

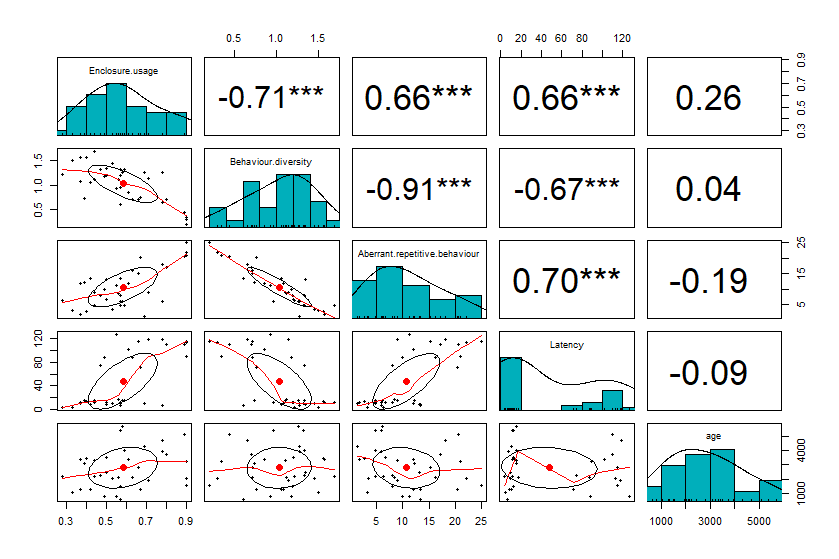

Supplement: Figure S2 — The ellipsoids inside each scatterplot represent 50% concentration of data points. The upper diagonal box represents correlation coefficients between welfare indices with significance values ∗0.05, ∗∗0.01, ∗∗∗0.001. [file peerj-08-8425-s002.png]
