# Supplementary material for: Effects of personality and rearing-history on the welfare of captive Asiatic lions (Panthera leo persica)
Source: PeerJ. 2020 Feb 6;8:e8425. doi: 10.7717/peerj.8425 (PMC7007979; doi:10.7717/peerj.8425)
Supplement: Table S1 [file peerj-08-8425-s003.docx]

Supplementary Table 1: Personality traits adapted from Chadwick, 2014 and Wielebnowski, 1999)

| Sl | Trait | Definition | Trait cluster |
| --- | --- | --- | --- |
|  | Active | Moves around enclosure (e.g. paces, runs, stalks) | Bold |
|  | Aggressive to conspecifics | Reacts hostile (e.g. attacks, growls) towards other lions | Shy |
|  | Aggressive to familiar people | Reacts hostile and threatening to familiar keepers and staff members | Shy |
|  | Aggressive to unfamiliar people | Reacts hostile and threatening towards unfamiliar staff and members of the public | Shy |
|  | Calm | Not easily disturbed by changes in the environment | Bold |
|  | Curious | Approaches and explores changes in the environment | Bold |
|  | Eccentric | Shows stereotypic or unusual behaviours | Shy |
|  | Excitable | Overreacts to changes in the environment | Bold |
|  | Friendly to conspecifics | Initiates and seems to seek proximity of other lions | Bold |
|  | Friendly to keepers | Initiates proximity with keepers: approaches fence readily and in a friendly manner (e.g. purrs, rubs on fence) | Bold |
|  | Fearful of conspecifics | Retreats and hides from other lions | Shy |
|  | Fearful of familiar people | Retreats and hides from familiar keepers and staff members | Shy |
|  | Fearful of unfamiliar people | Retreats and hides from unfamiliar staff and members of the public | Shy |
|  | Insecure | Seems scared easily; “jumpy” and fearful in general | Shy |
|  | Playful | Initiates and engages in play behaviour (seemingly meaningless, non-aggressive behaviour) with objects and/or other lions | Bold |
|  | Self-assured | Moves in a seemingly confident, well-coordinated and relaxed manner | Bold |
|  | Smart | Learns quickly to associate certain events and appears to remember for a long time | Bold |
|  | Solitary | Spends time alone; avoids company | Shy |
|  | Tense | Shows restraint in movement and posture | Shy |
|  | Vocal | Frequently and readily vocalizes | Bold |

Supplementary Table 2: Bold and shy behaviours used for novel object tests

| Behaviour | Description |
| --- | --- |
| Bold behaviours | |
| Approach | Subject approaches novel object confidently, or in a stalking stance |
| Play | Play with or solicit play with unknown conspecific |
| Sniff | Sniff the novel object cautiously |
| Interaction | Physical interaction with novel object (including destruction of the same) or showing the urge to interact physically with unknown person by rubbing body by the cage. |
| Shy behaviours | |
| Cowl or avoid | Limit oneself at the corner of the cell to maximize distance from novel object. |
| Run away | Sprint away from novel object |
| Vocalize | Growl at novel object without interacting with it |
| Aggression | Charging towards novel object combined with growling |
| Displacement | Perform autogrooming behaviours and avoid the novel object |

Supplementary table 3: Comparative table for the proportion of bold and shy behaviours performed by subjects during novel object tests

| **Group Statistics** | | | | | |
| --- | --- | --- | --- | --- | --- |
|  | Personality | N | Mean | Std. Deviation | Std. Error Mean |
| Shy behaviours | Shy lions | 14 | 84.14 | 9.591 | 2.563 |
|  | Bold lions | 21 | 12.76 | 8.746 | 1.909 |
| Bold behaviours | Shy lions | 14 | 15.86 | 9.591 | 2.563 |
|  | Bold lions | 21 | 87.24 | 8.746 | 1.909 |

Supplementary figure 1

Unscaled representative schematic layout of enclosures at the Asiatic lion conservation breeding programme at Sakkarbaug zoo that housed subjects during the study.

Supplementary figure 2

Exploratory data analysis of Scatterplot matrix (lower diagonal) with bivariate Pearson’s correlational analysis (upper diagonal) of various welfare indices viz., Enclosure usage, Behaviour diversity, Aberrant repetitive behaviour (ARB), Latency to novel objects, and age of subjects. The ellipsoids inside each scatterplot represent 50% concentration of data points. The upper diagonal box represents correlation coefficients ( Pearson’s) between welfare indices with significance values * 0.05, **0.01, ***0.001.
